# Physiological and economic benefits of abandoning invasive surgical procedures and enhancing animal welfare in swine production

**DOI:** 10.1038/s41598-019-52677-6

**Published:** 2019-11-06

**Authors:** Liat Morgan, Beata Itin-Shwartz, Lee Koren, Jerrold S. Meyer, Devorah Matas, Ahmad Younis, Shiri Novak, Nathalie Weizmann, Olja Rapaic, Weissam Abu Ahmad, Eyal Klement, Tal Raz

**Affiliations:** 1 Koret School of Veterinary Medicine, Robert H. Smith Faculty of Agriculture, Food and Environment, The Hebrew University of Jerusalem, Rehovot, 761001 Israel; 2 Environmental Economics and Management, Robert H. Smith Faculty of Agriculture, Food and Environment, The Hebrew University of Jerusalem, Rehovot, 761001 Israel; 3 Faculty of Life Sciences, Bar-Ilan University, Ramat-Gan, 5290002 Israel; 4 Department of Psychological and Brain Sciences, University of Massachusetts Amherst, Amherst, MA 01003 USA; 5 Lahav C.R.O, Comprehensive Pre-Clinical Services, Lahav, 85335 Israel

**Keywords:** Reproductive biology, Zoology

## Abstract

Food-animal welfare is a major ethical and social concern. Pork is the most consumed meat worldwide, with over a billion pigs slaughtered annually. Most of these pigs routinely undergo painful surgical procedures (surgical castration, tail docking, teeth clipping), which farmers often reluctant to avoid, claiming it would increase cost and reduce production efficiency. Herein, this study indicates that these procedures compromise pigs’ health and condition. Replacing surgical castration with immunocastration, avoiding tail docking and teeth clipping, and providing environmental enrichment, resulted in significant increase in weight gain, lowered risks for injuries and death, and reduced saliva and hair cortisol, both biomarkers for stress. Testosterone and DHEA analyses confirmed that immunocastration was an effective alternative to surgical castration. Economic models for the entire US swine market revealed that following across-the-board acceptance of this management, pork meat price is expected to drop, while the total annual social welfare (combined consumer and producer surplus) is expected to increase by $US 1.48 to 1.92 billion. In conclusion, sustainable swine farming management can be beneficial for both animals and farmers. Applying such welfare-friendly management is expected to reduce stress, enhance piglet/pig welfare and production, and improve the economics of swine operations in the global agro-food system.

## Introduction

The welfare of food animals is a growing concern. Farming of animals under human care is no longer seen as merely a means of food production, but also as an ethical concern, which also has economic implications^1–3^. Livestock farming is under increasing pressure to become more and more efficient in order to meet the demands of feeding the estimated world population of 9.7 billion by 2050^4,5^. Therefore, farmers and producers are often reluctant to accept some policies intended to improve animal welfare, claiming it will increase production costs and reduce production efficiency. Pork is the most consumed meat worldwide, with a global industry that includes more than a billion pigs slaughtered annually^6,7^. In most countries, piglets routinely undergo a set of invasive procedures during the first days of their lives. These procedures commonly include surgical castration and tail docking, and in many countries also teeth clipping^8–11^. Surgical castration is performed in order to reduce aggressiveness and eliminate “boar taint”, an unpleasant odor of the meat that results from the accumulation of skatole and androsterone after puberty in uncastrated pigs^12–14^. Surgical castration includes a scrotal incision, a blunt dissection of the testis from the surrounding fascial tissue, and pulling of the spermatic cord until it ruptures or is cut^15^. One of the alternatives is immunocastration by an anti-GnRH vaccine, which has been shown to prevent boar taint^12–14^.

Tail docking aims to reduce the incidence and damage caused by tail biting by other pigs^15^. It is done by a partial tail removal using cautery irons, clippers, scalpels, or scissors. Teeth clipping is used to minimize potential bite injuries to other pigs, as well as to prevent sow teat injuries^16^. It is done by grinding the canine teeth or removing them. In many countries, these surgical procedures are commonly performed without anesthetics, and at the same time, at the age of a few days. Each of these invasive procedures involves a degree of tissue damage, resulting in pain and short- and long-term stress, which may have negative effects on the piglets’ health, welfare, and production parameters^11,17^.

Pigs are considered to be one of the most intelligent animals, with natural behavior of foraging and exploring. When meaningful environmental enrichment is not provided, as well as other factors such as low space allowance, the manipulative behavior is commonly directed to other pen-mates. This mouth manipulation behavior includes tail biting, and may lead to other injuries and additional stress^10,18^. Moreover, apart from the prevention of undesired aggressiveness between pen-mates, there are other advantages for providing environmental enrichment; it improves maternal behavior, cognitive performance of the piglets, as well as their weight gain^19–24^. It has been also suggested that the lack of environmental enrichment at an early age may cause chronic stress to pigs, which might impair the cortisol circadian rhythm and reduces the hypothalamic-pituitary-adrenal (HPA) axis response to stress^25^.

Cortisol is a glucocorticoid produced by the adrenal cortex in response to adrenocorticotropic hormone (ACTH) secretion. It is considered an indicator of the body’s hormonal responses to stress, and is regulated by the HPA-axis. Cortisol can be measured in blood, urine, saliva, and recently also in hair samples. Hair analysis has increasingly been used, in human and in some animal species, as a non-invasive method to obtain information on long-term HPA-axis activity, for the evaluation of chronic stress with a negligible influence of acute stress^26^. Interestingly, several authors have reported on the relationship between altered physiologic status and high cortisol concentrations in blood and hair^27–30^. However, there is a lack of information regarding hair and saliva cortisol concentrations in piglets after surgical castration, tail docking, and teeth clipping. Furthermore, there is a need to critically evaluate welfare-friendly alternatives to these procedures, such as the use of immunocastration by an anti-GnRH vaccine as an alternative to surgical castration, and the possible benefits of adding environmental enrichment. Lastly, there is a need to critically compare the economics of conventional management, to the alternatives, so that producers, stakeholders, and legislators can consider applying welfare-friendly alternative management practices.

Therefore, the objective of the current study was to compare welfare physiologic and physical parameters, as well as production performances, of piglets from birth to slaughter, when surgical castration is replaced by anti-GnRH immunocastration, and tail docking and teeth clipping are avoided; as compared to conventional management, in which the three invasive procedures are performed, with and without meaningful environmental enrichment. A further aim was to construct economic models in order to estimate whether the proposed welfare-friendly rearing method can be profitable for producers at the farm level, as well as for the entire US swine industry in market equilibrium. All comparisons were made while pigs were under similar space conditions (based on the EU directive recommendations) and housed on slatted floor.

## Results

In this study, production and welfare parameters were measured under different management practices from birth to slaughter. Overall, all production and welfare measurements significantly improved when invasive procedures were avoided and environmental enrichment was provided. Furthermore, our economic model revealed a substantial expected increase in profitability following these changes, at the farm level, as well as for the entire US swine market.

### Production and survival parameters

**Figure 1:**
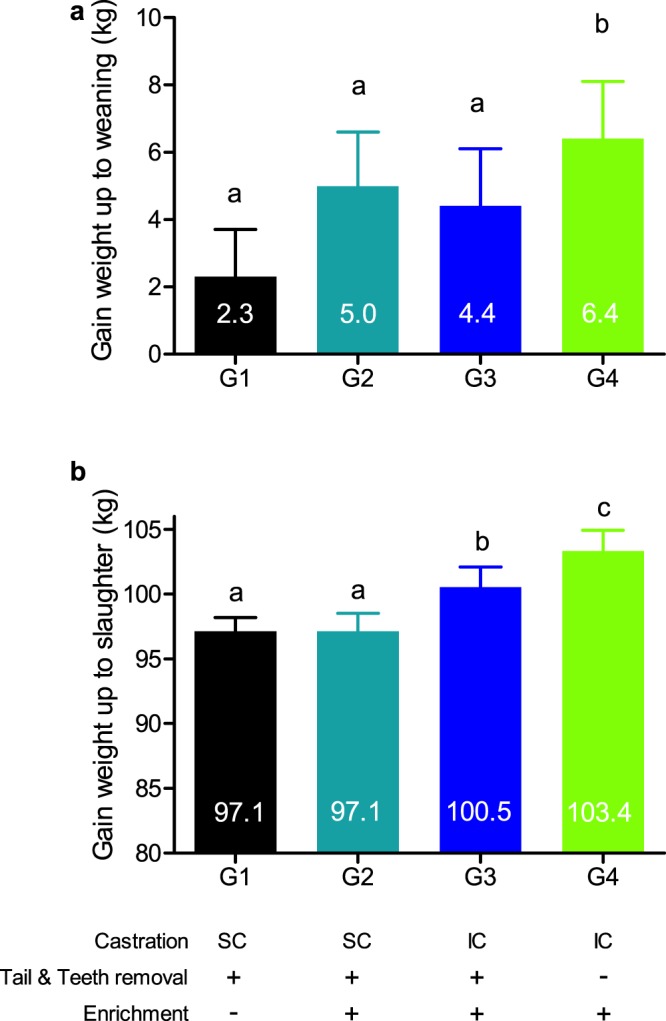
Piglets/pigs gained body weight up to weaning and slaughter, as husbandry invasive surgical procedures were gradually avoided and meaningful environmental enrichment was provided. Body weight differences from birth to weaning (**a**) and (**b**) slaughter were analyzed by a mixed effect linear regression model. Random effect: mother. Predictor: treatment group (G1-4). Adjusted for: gender, sows’ cycle number, the number of raised piglets, suckling period length and group score given by the farmer on weaning day. ^a,b,c^ Columns with different letters differ significantly (*P* < 0.05).

In general, production and survival parameters were significantly higher when invasive procedures were avoided (Groups 3,4). Mixed-effects linear regression models revealed that although there were no significant differences in birth weight among groups, as well as no significant differences in male:female ratios among the groups, weaning weights as well as slaughter weights were significantly higher when invasive procedures were avoided (see Fig. 1 and Supplementary Table S2). When only surgical castration was avoided (Group 3), the adjusted slaughter weight (slaughter weight minus birth weight) was higher by 3.4 kg per pig (*P* = 0.035), as compared to the conventional Group 1. Furthermore, when all invasive procedures were avoided (Group 4), adjusted weaning weight increased by 4.1 kg (*P* = 0.040; Fig. 1a) and slaughter weight increased by 6.3 kg per pig (*P* < 0.001; Fig. 1b).

**Figure 2:**
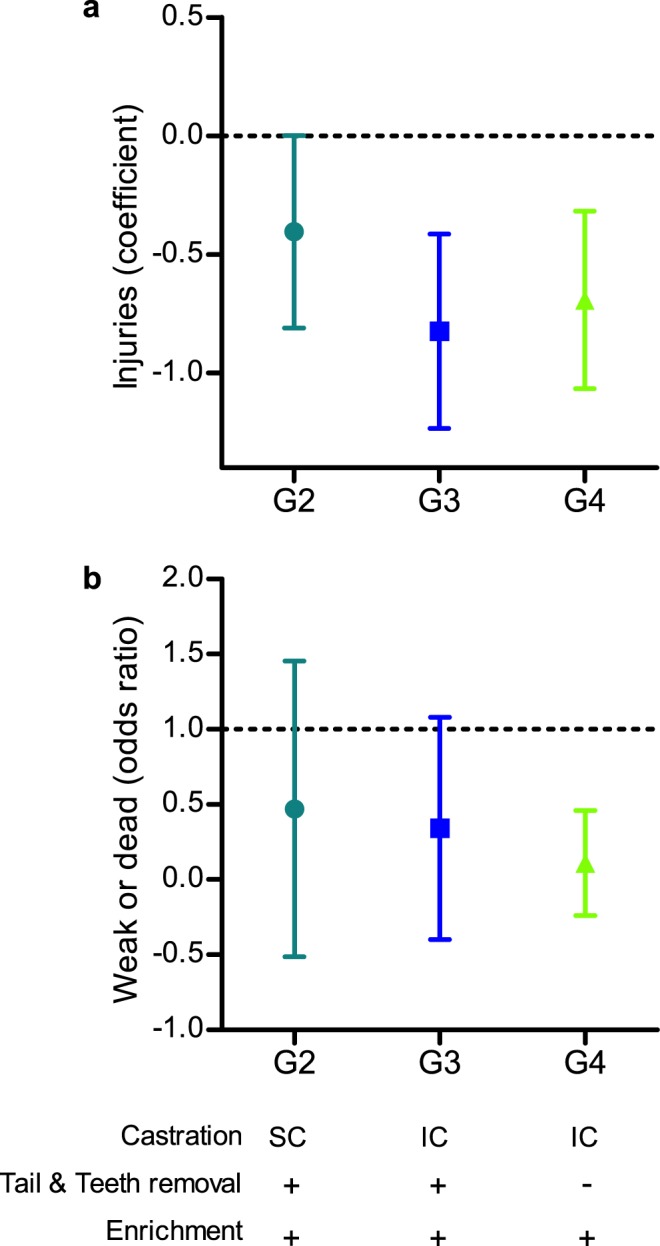
Injuries, weakness and mortality of piglets/pigs as husbandry invasive surgical procedures were gradually avoided and meaningful environmental enrichment was provided. (**a**) Mixed effects Poisson Regression Coefficient analysis of injuries occurrence in Groups 2-4 (G2-4) as compared to the control group (Group 1; G1); data is presented as coefficient (sign) and 95% confidence interval (bars). (**b**) Odds Ratio to be weak or dead in Groups 2-4 (G2-4) as compared to the control group (Group 1); data was analyzed by a chi-square goodness of fit test and the Binominal test, and is presented as Odds Ratio (sign) and 95% confidence interval (bars). In both panels, Group 1 is represented by the horizontal dotted line; values significantly differed (*P* < 0.05) from Group 1 if the bar does not cross the dotted line.

The total numbers of dead, weak, and injured piglets and pigs (lame, tail bitten or with skin injuries) were overall low in this study, but were more common among the conventional, non-enriched Group 1, compared to the other groups (Fig. 2; Supplementary Tables S3–S5). In addition, the odds ratio for weak or dead piglets/pigs in Group 1 was 89% higher than in Group 4, in which all invasive procedures were avoided and meaningful environmental enrichment was provided (*P* = 0.015, Fig. 2b; Supplementary Table S4). Interestingly, tail biting occurred only in Groups 1 and 2, where tail docking was performed, and although it was overall infrequent in this study, it was more common when environmental enrichment was not provided and invasive procedures were performed.

### Physiological welfare measures

**Figure 3:**
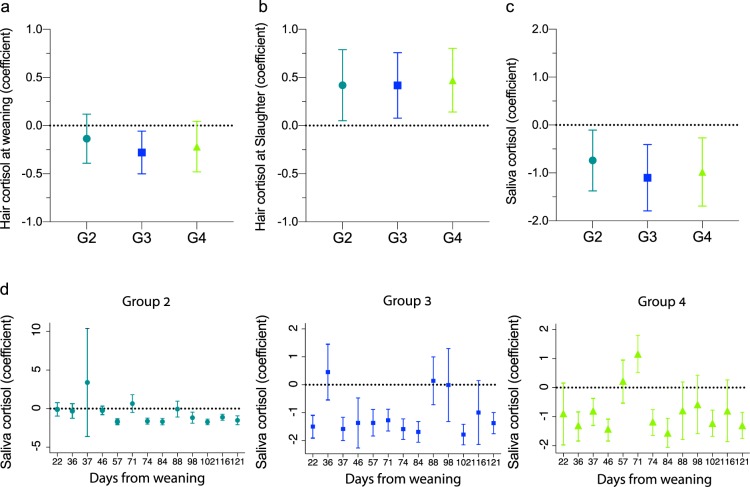
Hair and saliva cortisol concentrations in piglets/pigs, as husbandry invasive surgical procedures were gradually avoided and meaningful environmental enrichment was provided. Individual hair cortisol concentrations at weaning (**a**) and slaughter (**b**) were analyzed by Multi Variate Linear regression test (controlled for variables: mother cortisol, weaning weight and suckling period). Saliva was collected for cortisol measurements at the pen level, every two weeks from weaning to slaughter, and the results are presented in (**c**) and (**d**). (**c**) Samples were clustered together per group, without taking the sample date into consideration; a mixed effect linear regression model was used for statistical analysis (Random effect: pen. Predictor: treatment group. Adjusted for: tail biting, leg injuries, skin lesions, weakness and survival). (**d**) Analysis of saliva cortisol in Group 2-4, as compared to Group 1; for the statistical analysis, time varying group effects was applied, using log-level regressions on group identifiers and individual treatments, standard errors were clustered at the pen level. In all panels, data is presented as coefficient (sign) and 95% confidence interval (bars); Group 1 is represented by the horizontal dotted line; values significantly differed (*P* < 0.05) from Group 1 if the bar do not cross the dotted line.

Previous studies have shown that hair cortisol reflects exposure over long periods of time, and therefore, hair cortisol concentration can be used as a potential biological marker for chronic stress^31–33^. In the current study, hair cortisol from birth to weaning (hair sampling upon weaning), tended to be lower when invasive procedures were avoided and environmental enrichment was provided. In Group 3, where surgical castration was avoided and environmental enrichment was provided, hair cortisol at weaning was significantly lower by 32.2% (*P* = 0.014, Fig. 3a); and in Group 4, when all invasive procedures were avoided, hair cortisol was lower by 24.5% (*P* = 0.102; Fig. 3a), compared to Group 1. Moreover, the analysis revealed that higher weaning weight was significantly associated with decreased hair cortisol by 6.64% per additional kilogram of weaning weight (*P* < 0.001).

From weaning to slaughter, cortisol was measured in saliva, every two weeks, as a potential biological marker for acute stress, at the pen, as described in Methods. The measured concentrations of saliva cortisol were comparable with previous reports^34,35^. In most samples, saliva cortisol was significantly lower when meaningful environmental enrichment was provided (Groups 2, 3, and 4) as compared to Group 1 (*P* < 0.05; Fig. 3d). When all samples were clustered together per group (without considering the sampling dates), saliva cortisol was significantly lower in all enriched groups, as compared to the conventional, non-enriched Group 1 (Group 2: −0.74 ± 0.37, Group 3: −1.1 ± 0.35, Group 4: −0.98 ± 0.36 ng/mL as compared to Group 1; *P* ≤ 0.011; Fig. 3c, Table S6).

Cortisol concentration was also measured in hair sampled from the pigs a day before slaughter. It was significantly higher in Groups 2–4 compared to Group 1. Interestingly, our analysis also revealed that hair cortisol concentrations of pigs at slaughter were predicted by the hair cortisol concentration of the sow at the time of weaning; pig hair cortisol at slaughter significantly increased by 0.483% for an increase of 1% in sow’s cortisol at the time of weaning (*P* < 0.01; Fig. 3b).

### Testosterone and Dehydroepiandrosterone in males

**Figure 4:**
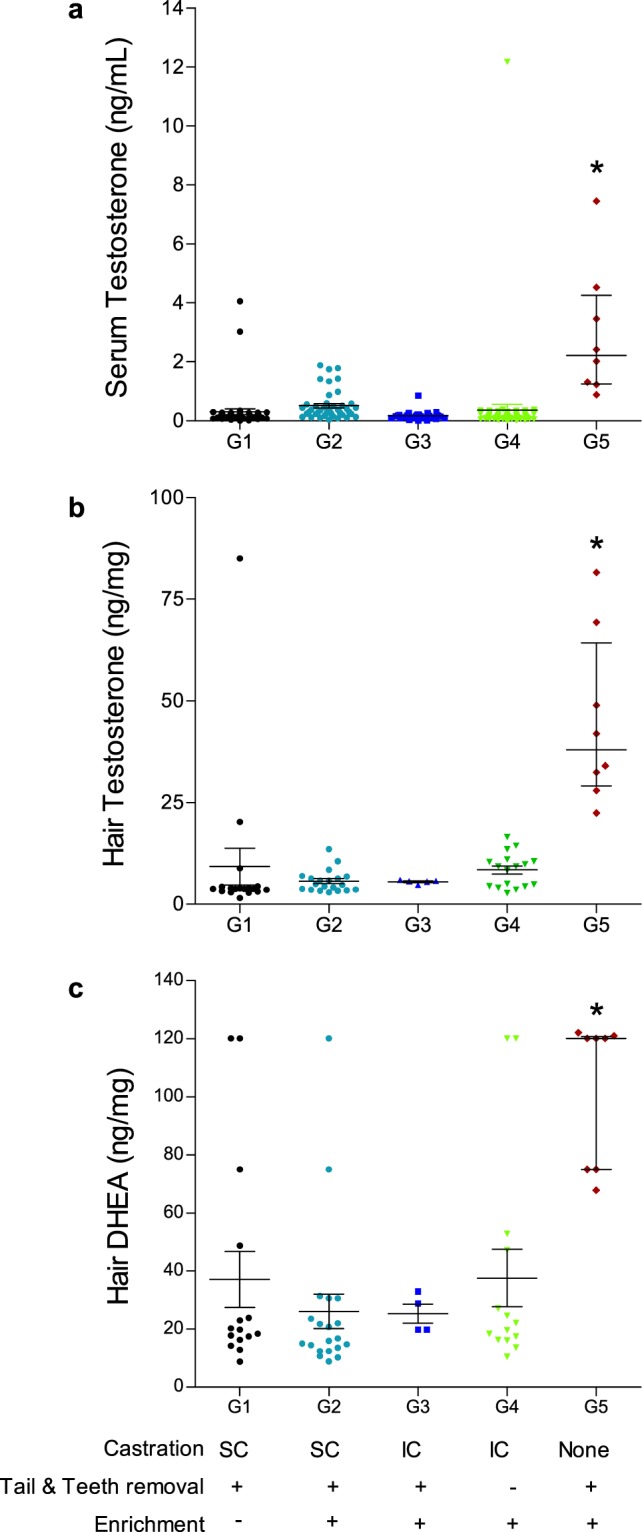
Testosterone and Dehydroepiandrosterone (DHEA) concentrations in male pigs at slaughter, following surgical castration, immunocastration, and in intact males. Serum Testosterone (**a**), hair Testosterone (**b**) and hair DHEA (**c**) concentrations were compared among groups by Kruskal-Wallis test, followed by Bonferroni test: surgically castrated males (SC: G1&2), immunocastrated males (IC: G3&4), and intact males (G5). *P < 0.05.

In order to assess whether puberty was delayed by the anti-GnRH vaccine as an immunocastration alternative to surgical castration, testosterone and DHEA (Dehydroepiandrosterone) were measured in males at the end of the fattening period (just prior to slaughter). Testosterone concentrations in the serum and hair were compared between the two non-surgically immuno-castrated groups (Groups 3 & 4), to the surgically castrated groups (Groups 1 & 2), as well as to 8 entire male pigs from the same age (Group 5). Testosterone concentrations in both serum (Fig. 4a) and hair (Fig. 4b) were similar for all kinds of castrations (SC; Surgical. IC: immunocastration); as expected, they were significantly lower compared to Group 5 of intact males (Fig. 4; *P* < 0.05). Similarly, hair DHEA levels were also significantly lower in surgically castrated and immunocastrated males compared to intact males (*P* < 0.05; Fig. 4c).

### Economic model for expected profitability

An economic model was constructed in order to examine whether the proposed welfare-friendly alternative to the husbandry invasive procedures can also be profitable for swine producers. The potential annual economic impact of shifting from the conventional rearing to the welfare-friendly rearing were assessed (i) for a small swine farm of approximately 12,000 slaughtered pigs per year, and (ii) for the entire US swine market. The model considered differences in rearing costs (equipment, manpower, materials), differences in slaughter weight, and differences in survival rates (Table S7). Overall, the economic model revealed a decrease in production costs when avoiding surgical procedures (as illustrated in the Methods and Table 1); the resulting decrease in labor costs was $1.65 per head. For the conventional rearing, the calculated cost of pain relief drugs and equipment necessary for the invasive procedures was $0.5617 per head, which can be saved by avoiding the surgical procedures; for the alternative rearing method, the cost of the anti-GnRH vaccine was added ($1.6648 per male pig). Differences between the conventional rearing and the welfare-friendly rearing in slaughter weight (Conventional: Average: 97.1 kg; 95%CI: 94.94 to 99.26 kg. Welfare-friendly: 103.4 kg; 100.26 to 106.54 kg) as well as in mortality rates (Conventional: 14%. Welfare-friendly: 5%) were included in the model based on the results of the current study data (Table S7). The cost of “extra-raising” (i.e., raising additional pigs in order to reach an aspired total number of pigs reaching slaughter) was calculated as the rate of mortality multiplied by the average cost of rearing, which was reported to be approximately $120 per head by the USA National Pork Board^36^. Combining these items yielded that avoiding the surgical procedures and applying immunocastration is expected to decrease the cost of rearing by $0.1375 per kg of market hog (Table S7; Table 1).

**Table 1:** Expected differences in costs of production, meat quantities sold and profits for a small farm (12,000 pigs) in conventional rearing as compared to welfare-friendly rearing.

	Conventional rearing	Welfare-friendly rearing	Difference
Number of slaughter pigs	12,000	12,000	0
Expected slaughter weight (kg/pig)	97.1 [94.944; 99.256]	103.4 [100.264; 106.536]	6.3 [2.476; 10.124]
Meat price ($US per kg)	2.2	2.2	0
Production cost ($US per kg)	0.2045 [0.201; 0.208]	0.0670 [0.065; 0.069]	−0.1375 [−0.142; −0.133]
Total sold meat quantity (Thousand kg)	1,165 [1,144; 1,187]	1,241 [1,209; 1,272]	76 [37.26; 113.9]
Total revenue ($US)	2,566,520 [2,518,732; 2,614,428]	2,733,040 [2,663,422; 2,802,724]	166,520 [82,081; 250,894]
Total cost ($US)^#^	238,200	83,070	−155,130
Total Profit ($US)^#^	2,328,320 [2,280,533; 2,376,229]	2,649,970 [2,580,350; 2,719,652]	321,650 [237,208; 406,021]

^#^Calculation excluding common costs. 95% Confidence intervals appear in squared brackets, as calculated using a bootstrap procedure.

Using this model, a profit calculation was constructed for a farm aiming to produce 12,000 slaughtered pigs per year (similar to the farm used in the current study). In this calculation, the meat price was held fixed, assuming that the farm is small in the sense that it has no effect on the market prices. Thus, as illustrated in Table 1, shifting from conventional to welfare-friendly rearing is expected to result in an annual higher sold meat quantity (total of +76,000 kg; 95%CI: 37,265 to 113,906 kg), a higher revenue (total of +$166,520; 95%CI: $82,081 to $250,894), with lower costs (total reduction of $155,130) and higher profits (total increase of +$321,650; 95%CI: $237,208 to $406,021) per year.

Next, the effect of across-the-board shift to the welfare-friendly rearing method for the entire US swine market was also calculated (Table 2). For this purpose, the equilibrium prices and meat quantities were calculated, to obtain the expected effect on profits. For the main analysis, the average supply and demand elasticities (0.417 and −1.078, respectively; Table S8) were used (results for the range of elasticities are presented in Table 3). The elasticities were coupled with current quantity and price statistics published for the US swine market, stating sold quantity of 9,463 million kg meat per year, for the price of $2.2 per kg^37^. Using this information, the full demand and supply equations could be backed out, assuming a linear form. The resulting supply and demand equations were:

$${Q}_{d}=19,\,664.182-0.005P$$

$${Q}_{s}=5,\,518.525+0.002P$$

Where *Q*_*d*_ is the quantity (demand) in 1,000 tons; *Q*_*s*_ is the quantity (supply) in 1,000 tons; and *P* is the price for 1,000 tons. The inverse supply curve under “welfare-friendly rearing” was shifted downwards by 0.1375 million $US, so the new supply curve $${Q}_{S}^{^{\prime} }$$ is:

$${Q}_{S}^{\text{'}}=5,764.72+0.002P$$

**Table 2:** Expected differences in equilibrium meat price and quantities as well as profits for the entire US market in conventional rearing as compared to welfare-friendly rearing.

	Conventional rearing	Welfare-friendly rearing	Difference
Meat price ($US per kg)	2.203 [2.203; 2.203]	2.164 [2.163; 2.165]	−0.038 [−0.039; -0.037]
Total sold meat quantity (Million Kg)	9,463.032 [9286.834; 9639.673]	9,640.576 [9465.919; 9815.686]	177.54 [174.87; 180.33]
Total revenue (Million $US)	20,843.5 [20,456; 21,233]	20,865 [20,477; 21,254]	21.5 [21; 21.7]
Total costs (Million $US)^#^	1,935 [1,935; 1,935]	645 [625; 665]	−1,290 [−1,310; -1,269]
Total profit (Million $US)^#^	18,909 [18,521; 19,298]	20,220 [19,843; 20,598]	1,311 [1,291; 1,331]
Consumer surplus (Million $US)	9,668 [9488; 9848]	10,034 [9857; 10211]	366 [361; 372]

^#^Calculation excluding common costs.

95% Confidence intervals appear in squared brackets, as calculated using a bootstrap procedure.

**Table 3:** Expected change in producer surplus, consumer surplus and total welfare when conventional rearing is replaced by welfare-friendly rearing, considering the possible ranges of demand and supply elasticities values. The minimum and maximum demand and supply elasticities values were according to the published literature, as detailed in Table S8.

	Demand elasticity				
	Min -0.5	Aver -1.08	Max -2.75		
Producer surplus change (billion $US)	1.1061 [1.089; 1.124]	0.9878 [0.973; 1.003]	0.9233 [0.909; 0.938]	Min 0.2	Supply elasticity
	1.3080 [1.288; 1.329]	1.3106 [1.291; 1.331]	1.3108 [1.291; 1.331]	Aver 0.42	
	1.4469 [1.425; 1.47]	1.5824 [1.558; 1.607]	1.6821 [1.657; 1.709]	Max 0.6	
Consumer surplus change (billion $US)	0.3734 [0.3677; 0.3793]	0.5957 [0.5866; 0.6051]	0.7156 [0.7047; 0.727]	Min 0.2	
	0.2047 [0.2016; 0.2079]	0.3662 [0.3606; 0.372]	0.4708 [0.4635; 0.4783]	Aver 0.42	
	0.0887 [0.0874; 0.0901]	0.1732 [0.1705; 0.1759]	0.2366 [0.2329; 0.2404]	Max 0.6	
Total social welfare change (billion $US)	1.4795 [1.457; 1.503]	1.5835 [1.56; 1.608]	1.6389 [1.614; 1.665]	Min 0.2	
	1.5127 [1.49; 1.537]	1.6767 [1.651; 1.703]	1.7816 [1.755; 1.81]	Aver 0.42	
	1.5356 [1.512, 1.56]	1.7556 [1.729; 1.783]	1.9187 [1.889; 1.949]	Max 0.6	

95% Confidence intervals appear in squared brackets, as calculated using a bootstrap procedure.

Thus, the expected economic results of shifting from conventional to welfare-friendly rearing, by avoiding the husbandry invasive procedures and applying immunocastration, for the entire US swine market, are summarized in Table 2. Pork meat price is expected to drop following the reduction in costs of raising, by $0.038 per kg (95%CI: −0.039 to −0.037 $US per kg). Sold meat quantities are expected to rise by 177.54 (95% CI: 174.87 to 180.33) millions kg per year. Sector revenues are expected to increase by 21.5 (95%CI: 21 to 21.7) million $US. Sector costs are expected to drop by 1,290 (95% CI: 1,310 to 1,269) million $US, and sector profits are expected to increase by 1,311 (95% CI: 1,291 to 1,331) billion $US, per year. Consumer surplus is also expected to grow by 366 (95% CI: 361 to 372) million $US. In addition, using the average supply and demand elasticities from the literature, total social welfare is expected to grow by 1.6767 (95% CI: 1.651 to 1.703) billion $US per year.

When considering the bottom and top from the range of elasticities in the literature (Table S8), the change in producer surplus ranges from 0.9233 (95% CI: 0.909 to 0.938) to 1.6821 (95% CI: 1.657 to 1.709) billion $US, and the change in consumer surplus ranges from 0.0887 (95% CI: 0.0874 to 0.0901) to 0.7156 (95% CI: 0.7047 to 0.727) billion $US. Accordingly, the increase in total social welfare, which is the combined consumer and producer surplus, ranges from 1.4795 (95% CI: 1.457 to 1.503) to 1.9187 (95% CI: 1.889 to 1.949) billion $US (Table 3).

## Discussion

Food-animal welfare is an issue of great importance worldwide^3^. For many consumers, the well-being of farm animals is strongly connected to the quality, and even safety of food, and this may change their consumption preferences^2,38,39^. However, actions in regard to animal welfare should be based on careful scientific studies, taking into consideration production and welfare parameters, including physiological parameters that are measured objectively; as well as the economic impacts on stakeholders and consumers. Still, in most countries around the world, piglets routinely undergo invasive surgical procedures during the first days of their lives, which commonly include tail docking, teeth clipping and surgical castration; these procedures can potentially cause pain and stress to the piglets, and may affect their short- and long-term welfare and production^17,40^. Therefore, the goal of the current study was to examine the economic, production and welfare implications of practical alternatives to the invasive procedures in piglets. Accordingly, we evaluated the production and welfare parameters of piglets from birth to slaughter when surgical castration, tail docking and teeth clipping were avoided, as compared to conventional management, in which all the invasive procedures were performed, with and without meaningful environmental enrichment. Several treatment groups were compared, in order to examine the added beneficial value of each of the main management changes. Overall, production and welfare parameters improved when invasive procedures were avoided, and environmental enrichment was provided. Immunocastraion was an effective alternative to surgical castration in delaying puberty. Furthermore, our economic model suggested an expected profitability following such changes, at the farm level, as well as for the entire US swine market. Therefore, the main conclusion from this study is that the suggested welfare-friendly alternatives reduce piglets/pig stress and can be economic and effective for both the farmers and the animals.

Replacing surgical castration with immunocastration, avoiding tail docking and teeth clipping, and providing environmental enrichment, were associated with increased weight gain, as well as reduced rates of injured, weak or dead piglets/pigs during the nursing and fattening periods. From the data of this study, it appears that these procedures may have additive effects, and the best production results are achieved when all of them are replaced. According to the data presented, the invasive surgical procedures may negatively affect both weaning and slaughter weight. While it is obvious why slaughter weight is important to the industry, it should be emphasized that weaning weight is also a valuable measure, as previous studies showed that low weaning weight can significantly predict post-weaning mortality and unfavorable low slaughter weight^41^. In the current study, both weaning weights and slaughter weights were significantly higher when more welfare-friendly management was applied. This finding could provide motivation for farmers to change and accept policies towards better animal welfare management worldwide.

It is clear that invasive procedures are likely to cause pain to piglets, which may have short- and long-term physiologic effects. Indeed, avoiding these surgical procedures was associated with reduced hair cortisol concentrations at the time of weaning, which represents cortisol accumulation in the piglets’ hair during the nursing period. As far as we know, this has not been shown in hair cortisol before. Furthermore, during fattening, salivary cortisol measurements were typically lower in all enriched groups, as compared to the conventional group. Cortisol is regulated by the hypothalamic-pituitary-adrenal (HPA) axis, and is influenced also by the circadian rhythm. Thus, collection timing is critical, particularly when measured in saliva, urine or blood. Furthermore, the procedure of sample collection by itself, particularly blood, can cause a rapid notable increase in cortisol concentration due to acute stress^9–11^. Therefore, saliva sampling by ropes appears to be a much better choice than blood, as pigs typically like chewing the sampling cotton ropes, given as an environmental enrichment. Although, saliva collection followed a strict protocol, some variation among pens could be detected, as many environmental factors (noises, fights in the pen, arousal or fear due to pigs’ movement in the building, etc.) may possibly change saliva cortisol concentration. In contrast to saliva and blood cortisol, studies in other species have shown that hair cortisol is a proxy measure to the total retrospective activity of the HPA-axis over weeks or even months^42^. The data of saliva and hair cortisol were complementary, and overall indicated that avoiding tail docking and teeth clipping, replacing surgical castration with immunocastration, and providing environmental enrichment, are associated with reduced stress and improved production, compared to the conventional management.

In previous studies, it was suggested that in barren pens for weaners and finishers, chronic stress is high, resulting in impairment of the circadian rhythm and reduces HPA-axis response to stress, and therefore, low saliva cortisol levels^43^, at least from a certain age^34^. In the current study, we found significantly lower saliva cortisol during the fattening period during most sampled weeks, but higher hair cortisol before slaughter, in all three environmentally enriched groups, compared to the non-enriched conventional group. Allegedly, the results may seem to contradict each other, and are also not consistent with the results of hair cortisol at weaning. However, there are a few potential explanations for that contradiction. It has been suggested that chronic stress results in blunted circadian rhythm, but only after a certain age, which varies among studies^25,34^; this may explain the relatively high hair cortisol at the age of weaning (21–24 days) but low hair cortisol before slaughter (5–6 months) among the pigs in the conventional group. Another explanation to this difference is that hair cortisol at weaning is an indication for the nursing period including the performance of the invasive procedures. Prenatal conditions could possibly affect this period as well, while the only management difference was that Groups 2–4 were environmentally enriched a few days before the expected farrowing. On the contrary, hair cortisol just prior to slaughter is relevant for the previous weeks before slaughter but the exact period of time is unknown and may be influenced by different factors. Saliva cortisol concentrations were measured as point samples every two weeks during the whole fattening period and not only at the end. Previously, it was suggested that cortisol can be a welfare measure when a particular factor is known as a welfare implication of animals, such as mutilations^34^. The invasive surgical procedures may be considered as such; however, their possible effects on the resulting long-term stress at the end of the fattening period is less clear. Interestingly, our analysis indicated that sows’ hair cortisol concentration was a significant predictor for its piglets’ hair cortisol only before slaughter, although our expectation was that it would be associated with both weaning and slaughter hair cortisol concentrations of their offspring^35,44,45^. This suggests that piglets’ hair cortisol at weaning was influenced mainly by the experience, or lack of the painful invasive surgical procedures, while later, at slaughter, genetic influence may have been more pronounced, as it was previously described that cortisol and the HPA stress response can have a genetic background^46^. However, further studies are needed in order to elucidate that possibility.

Prolonged stress has been suggested as a potentially harmful and often undetected risk factor for chronic illness in human and other species^42^. The overall occurrence of death, injuries and weakness of piglets and pigs were low in this study. The low number may be explained by the fact that actions were taken to minimize aggression among pen mates in all groups: the space allowance was according to the EU directive^47^, and regrouping occurred only once, on the weaning day, as a litter (each two litters were grouped together), in order to decrease interactions with unfamiliar individuals, which is considered to be a major risk factor for injuries^48,49^. Furthermore, for three out of four treatment groups, meaningful environmental enrichment was provided. Interestingly, the occurrence of death, injuries and weakness of piglets/pigs was lower when invasive procedures were avoided and environmental enrichment was provided. This indicates that these invasive procedures, especially when carried out on pigs raised without environmental enrichment, are a great compromise of pig health and condition.

In this study, the anti-GnRH vaccine, was an effective alternative to surgical castration in males, as serum testosterone concentrations at the time of slaughter were significantly lower as compared to intact males, and similar to surgically-castrated males, in accordance with previous reports^12,14,46,50,51^. As expected, we found that hair testosterone and DHEA concentrations at slaughter in vaccinated males were similar to the hair concentrations in surgically castrated animals, but significantly lower as compared to the concentrations in intact males. Previous reports indicated that serum DHEA concentrations significantly increase following puberty in male, humans and pigs. Furthermore, it was reported that DHEA decreased rapidly after surgical castration of male pigs^52–54^. Therefore, the hormonal findings regarding testosterone and DHEA in the current study indicate that the steroid production capacity of the testes in anti-GnRH vaccinated male pigs was reduced, which supports the conclusion that immunocastration and delayed puberty were indeed achieved.

The production of swine products is a major industry, as pork is the most consumed meat worldwide. Accordingly, the economic implications of management modifications should also be examined. As a result of our economic analyses, we estimate that a producer aiming to raise 12,000 pigs to slaughter annually, can potentially gain approximately $320,000 in annual profits. The rise in profitability stems from reduced costs, increased survival rates, and higher slaughter weight, as have been shown in this study. When considering the market equilibrium changes for the entire US market, we found an expected decrease in meat price following the reduction in marginal costs of rearing. Furthermore, according to our model, sold quantities are expected to increase, total sector profit is expected to increase by approximately 1.3 billion $US (this equals to approximately 5.6% of reported sector revenues^55^) and the total social welfare is expected to rise by 1.48 to 1.92 billion $US. Furthermore, considering a variety of values for demand and supply elasticities relevant to the US pork market, results consistently showed beneficial effects for the welfare-friendly rearing, demonstrating the robustness of the effect. Clearly, these calculations were affected by the numbers and values inserted into the model, which were adjusted mainly to the US market. Variations in costs of drugs, labor and materials among different countries might change these calculations, as well adding environmental enrichment, in countries where it is not mandatory today. Furthermore, our economic model may not completely fit all swine rearing systems, which vary greatly among farms, since it does not take into consideration the costs of the initial actions needed in order to meet the minimal EU directive standards, as well as during the transition to the suggested rearing system. Still, this economic model provides valuable information when considering improvement of swine welfare and economics. Therefore, at the farm level, it is advised that the suggested rearing system be carefully and specifically examined prior to implementation of such management (practically and economically).

At the market level, our economic model did not take into consideration two factors that are likely to influence economic benefits when producing pork under welfare-friendly management. First, we did not explicitly model the position of the US pork export in the global pork market following such change; however, we suggest that this is most likely expected to improve, due to the anticipated reduction in meat prices. Second, we did not consider the potentially higher “willingness to pay” on the side of consumers, for welfare-friendly meat, which potentially may increase profits even further. Previous studies have shown that consumers are willing to make an extra effort to buy animal welfare-friendly products, even if this means changing where they shop, or paying more for goods^56–58^.

In summary, this study indicates that replacing surgical castration by anti-GnRH vaccine, avoiding tail docking and teeth clipping, as well as providing environmental enrichment, is expected to be beneficial for both the farmers and the animals. Applying such welfare-friendly and relatively simple management is expected to reduce stress, enhance piglet/pig welfare and production, and improve the economics of swine operations in the global agro-food system.

## Materials and Methods

### Animals and study design

The study was conducted during 2017, at Lahav Animal Research Institute (LAHAV) and the Hebrew University, according to the ethical approval of the Hebrew University’s Institutional Animal Care and Use Committee (MD-16-14754-2). The study included sows (n = 32) from parities 2–8, mixed breed of Landrace, Large-White, Pietrain and Duroc, and their piglets (n = 329 alive piglets on the day invasive procedures were performed), individually identified by ear tags. Sows and their litters were randomly and simultaneously assigned to four treatment groups (Supplementary Table S1); there were no significant differences in litter size (ranged from 9–13 piglets/litter), or males to females ratio among the groups (% male ranged from 47% to 53%). **Group 1** (n = 8 litters, 88 piglets): Surgical castration, tail docking and teeth clipping were performed (Meloxicam was injected a few minutes before the procedure) on the third day of their lives, without additional of environmental enrichment; **Group 2** (n = 8 litters, 82 piglets): same as Group-1, but meaningful environmental enrichment was provided; **Group-3** (n = 8 litters, 78 piglets): tail docking and teeth clipping were performed at the age of three days, piglets were not castrated but were vaccinated later in their lives with anti-GnRH vaccine, and environmental enrichment was provided; **Group 4** (n = 8 litters, 81 piglets): none of the invasive husbandry procedures was performed, piglets were vaccinated with anti-GnRH vaccine, and environmental enrichment was provided. A fifth group of 8 intact males from the same age group was sampled once (hair and blood samples) at the age of slaughter (**Group 5;** about 145 days old). The anti-GnRH vaccine (Improvac®, Zoetis) was administrated according to the the two-dose administration manufactur’s protocol; 2 mL subcutaneous injections were perfoemed twice; the first at 9–10 weeks of age (about 70 days old), and the second at 4–5 weeks prior to slaughter (at approximetly 110 days old).

### Housing and environmental enrichment

Animals in all treatment groups were kept under the same conditions during the different stages of rearing (gestation; farrowing and nursing; fattening). Solid commercial swine food (Ambar Feed Institute, Israel) was provided by electronic automatic feeders according to the recommendations of the Nutrient Requirement (NRC) of swine. Approximately 4–5 days prior to anticipated farrowing, sows were transferred into individual farrowing crates. Sows were kept in farrowing crates for 10 days and then moved to designed pens, in which the sow and her piglets were free in their open crates until weaning (21–24 days). For environmental enrichment, in Groups 2–4, cotton ropes and jute strips were hung from the crates/pen bars, starting a few days before anticipated farrowing, and were refilled during the whole lactation period (Supplementary Fig. S1). The cotton ropes and jute strips were placed close to the sow’s head. Piglets started chewing the materials, following their mothers, usually at the age of 2–3 days.

On weaning day, two litters from the same treatment group were grouped into one pen group that remained static throughout the fattening period, until slaughter (groups of 16–21 pigs); space allowance was similar among groups and was based on the EU directive^47^. As environmental enrichment, until the age of 70 days, cotton ropes and Bite-Rite® chewable silicone sticks devices (Ikadan System, Denemark) were provided (Supplementary Fig. S1); cotton ropes and chewable silicon sticks of the Bite-Rite® devices were maintained constantly. From the age of 70 days to slaughter, each pen group was moved to another building (same building for all groups); environmental enrichment during the last period included straw, which was provided in commercial grubbing straw racks (Domino, Denmark) designed for pigs, as well as Bite-Rite® devices.

### Collection of data regarding production, survival and physical condition

Production parameters were measured and recorded by Farm® (Agrovision), including: parity; farrowing date; litter size; birth weight; weaning date; weaning litter score (subjective scoring of the farmer:1, homogenies group of big piglets; 2, homogenies group but 1–2 piglets are weaker/smaller; 3, heterogenic group, up to 50% of the piglets are weaker/smaller than the average; 4, more than 50% of the piglets are weak or small); individual weaning weight; weight on the day moving from weaners to finishers housing (at the age of approximately 70 days); slaughter weight; and date of death when occurred. In addition, body condition (injuries and lames) was evaluated every two weeks by an experienced professional, and included binary records regarding tail biting (0, No; 1, Yes), weakness (0, No; 1, Yes), illness (0, No; 1, Yes), lameness (0, No; 1, Yes), and skin lesions (0, No or mild; 1, moderate to severe).

### Collection of hair, saliva and serum for Cortisol, Testosterone and Dehydroepiandrosterone analyses

Hair was clipped from each piglet on weaning day (just prior to weaning) and one day before slaughter. It was also clipped from the sows on weaning day. Hair was collected from just above the tail, and each sample was individually stored in aluminum foil at −20 °C until steroid extraction and analysis. Hair cortisol extraction was performed and analyzed by commercial enzyme-linked immunosorbent assay (Salimetrics ELISA kit, catalogue no. 1-3002, Carlsbad, CA, USA), according to a validated protocol for pigs^59,60^; Intra- and inter-assay coefficients of variation were 6.1% and 3.9%, respectively. Testosterone and Dehydroepiandrosterone (DHEA) were extracted and analyzed by commercially enzyme-linked immunoassay (Salimetrics saliva ELISA kits, catalogue no. DHEA 1-1202, and Testosterone 1-2402, Carlsbad, CA, USA), according to the protocol of Koren *et al*.^61^. Intra- and inter- coefficient of variation were 11.86% and 15.3% for testosterone, and 1.3% and 10.78% for DHEA, respectively.

Blood samples were obtained during slaughter in serum tubes, centrifuged and serum stored in −80 °C until analysis of testosterone. Serum testosterone samples were measured in duplicates by enzyme-linked immunosorbent assay (ELISA; DRG Serum Testosterone Kit (Eia1559), DRG International Inc. NJ, USA). Intra- and inter coefficient of variation were 5.8% and 4.5%, respectively.

Saliva samples were collected every two weeks using cotton ropes (TEGO^™^ Swine Oral Fluids Kit, USA) for one pooled oral sample from a group pen per sampling day. Saliva sampling for cortisol measurement was carefully performed, under a strict protocol; at the same time of the day (25 minutes prior to the second feeding), and at the same time for all groups. Cotton ropes were hung in the pen for 25 minutes just prior to the second feeding on each sampling day. Saliva samples were extracted and kept in −80 °C until analyzed. Saliva cortisol samples were measured in duplicates by enzyme-linked immunosorbent assay (ELISA; DRG Saliva Cortisol Kit, Cat# Slv2930, DRG International Inc. NJ, USA). Intra- and inter coefficient of variation were 5.6% and 9.6%, respectively.

### Economic model

For the economic model, calculations were based on the cost and output differences as found in this study, coupled with additional data on the US pig market (Table S7). For the US market analysis, a “back of the envelope” market equilibrium calculation was applied, using demand and supply elasticities from the literature on the US pork market (Table S8). Costs were calculated based on the literature and the current study (Table S7). Labor costs were calculated for the difference in work hours (as reported by the study farmer), which went from two hours per 32 heads, to one hour per 180 heads, in the conventional and welfare-friendly managements, respectively (Table S7). The resulting change in labor costs was therefore $1.65 per head. Material costs calculation for the invasive procedure rearing method included expenses of pain relief and equipment necessary for the procedures. Pain relief is 0.005 Euro per male (Lidocaine) and additional 0.14 Euro per head (Meloxicam). Equipment costs (such as surgical equipment) were reported by the study farmer to be approximately $1350 per year, for farm size of 12,000 pigs, which is 0.1125 $US per head. For the vaccine rearing method, cost of the immunocastration was included as incurred in this study, which was $1.6648 per male. To calculate changes in cost per kg, the differences in average slaughter weight and mortality rates were considered, based on the results of the current study. To obtain confidence intervals for the calculated values, an empirical bootstrap procedure was conducted. One million sets of samples of the size of the original samples (75 and 77), were sampled from a normal distribution with the mean and standard deviation of the original samples of slaughter weight values. For each simulated sample, the average weights were calculated and used to calculate the outcome variables (quantities, profits, etc.). Then, for every sample of each outcome the difference between the simulated outcome and the outcome calculated using average weights was derived. The 5^th^ and 95^th^ percentiles of these differences were used to construct the confidence intervals around the means.

### Statistical analyses

Statistical analyses were performed using commercial statistical software (IBM SPSS Statistics, version 24.0; STATA, version 14.0). Normal distribution of each variable was examined by the testing that Skewness and kurtosis equal to zero, by Shapiro-Wilk’s test for normality and by the shape of the Normal Q-Q plot. Accordingly, differences among groups in testosterone (hair and serum) and hair DHEA concentrations were analyzed by the Kruskal-Wallis test, followed by Bonferroni test, in order to adjust for multiple comparisons and to control for Type I error. Hair cortisol concentrations were analyzed by Multi-Variate Linear regression (adjusted for variables: mother cortisol, weaning weight and suckling period). For saliva cortisol, time-varying group effect was applied, using log-level regressions on group identifiers and individual treatments; standard errors were clustered at the pen level. A chi-square goodness of fit test and the mixed effects binominal test were used to compare the prevalence of dichotomous outcomes between selected treatments (e.g.; weakness and mortality), while outcomes with a number of events during a period of time were analyzed by Mixed-effects Poisson regressions (e.g., injuries), where the random effect was the sow ID. A mixed-effects linear regression model for repeated measures design was used for weight analysis (Random effect: sow ID. Fixed-effect: treatment group. Adjusted for: piglet/pig sex, sows’ cycle, number of born alive piglets, suckling period length, and farmers’ group score for the litter), followed by post-hoc Bonferroni test. Descriptive statistics are given as Mean ± SEM, 95% confidence interval, or as frequency (n) with percentage (%), as applicable. A *P* < 0.05 was considered statistically significant. All reported P-values were based on two-tailed hypothesis.

## Supplementary information

Supp Tables S1-S8 and Figure S1

## Data Availability

All data generated or analyzed during this study is included in this article (and its Supplementary Information files).
