# Supplementary material for: Physiological and economic benefits of abandoning invasive surgical procedures and enhancing animal welfare in swine production
Source: Sci Rep. 2019 Nov 6;9:16093. doi: 10.1038/s41598-019-52677-6 (PMC6834556; doi:10.1038/s41598-019-52677-6)
Supplement: Supplementary file 1 — Supp Tables S1-S8 and Figure S1 [file 41598_2019_52677_MOESM1_ESM.docx]

**Supplementary information for the article:**

**Physiological and economic benefits of abandoning invasive surgical procedures and enhancing animal welfare in swine production**

Liat Morgan^a^, Beata Itin-Shwartz^b^, Lee Koren^c^, Jerrold S. Meyer^d^, Devorah Matas^c^, Ahmad Younis^e^, Shiri Novak^a^, Nathalie Weizmann^a^, Olja Rapaic^d^, Weissam Abu Ahmad^a^, Eyal Klement^a^, Tal Raz^a*^

^a^Koret School of Veterinary Medicine, Robert H. Smith Faculty of Agriculture, Food and Environment, The Hebrew University of Jerusalem, Rehovot, Israel 761001.

^b^Environmental Economics and Management, Robert H. Smith Faculty of Agriculture, Food and Environment, The Hebrew University of Jerusalem, Rehovot, Israel 761001.

^c^Faculty of Life Sciences, Bar-Ilan University, Ramat-Gan, Israel 5290002.

^d^Department of Psychological and Brain Sciences, University of Massachusetts Amherst, MA 01003, USA.

^e^Lahav C.R.O, Comprehensive Pre-Clinical Services, Lahav, Israel 85335.

*Corresponding author (email address: tal.raz@mail.huji.ac.il)

**Table S1: Study design and treatment groups**.

Alive piglets at the age of three days were allocated randomly and simultaneously into four treatment groups (Groups 1-4) as a litter (8 litters per group). Treatments included either surgical castration or immunocastration with an anti-GnRH vaccine (Improvac®; for males only); tail docking and teeth clipping procedures were performed in some groups; and meaningful environmental enrichment was provided to all groups, excluding Group 1 (control conventional group). Piglets were raised by their mothers until weaning (2124-d). On the weaning day each two litters from the same treatment group were mixed into one housing pen for weaners. Groups remained static until slaughter. For some hormonal analyses, blood and hair samples were also collected from intact males (Group 5) at the age of slaughter.

| Environmental enrichment | Teeth clipping & tail docking | Mode of 'Castration' | n^#^ | Group |
| --- | --- | --- | --- | --- |
| No | Yes | Surgical Castration | 88 piglets | **1** |
| Yes | Yes | Surgical Castration | 82 piglets | **2** |
| Yes | Yes | Anti-GnRH vaccine | 78 piglets | **3** |
| Yes | No | Anti-GnRH vaccine | 81 piglets | **4** |
| Yes | Yes | None | 8 piglets | **5** |
| # n represent the total number of piglets in each group. There was no significant difference in litter size (ranged from 9-13 piglets/litter), or males to females ratio (%male ranged from 47% to 53%) among Groups 1-4. | | | | |

**Table S2:** **body weight gain from birth to weaning (21-24d), age of 70d, and slaughter.** Data was analyzed by a mixed-effects linear regression model for repeated measures design [Random effect: mother. Predictor: treatment group. Adjusted for: gender, sows’ parity, Number of raised piglets (alive piglets on the day performing invasive procedures), suckling period length and group score given by the farmer on weaning day]. Standardized gained weight (kg) and standard error mean (SEM; in brackets) are presented (upper panel). Results of post-hoc analysis for differences between groups are represented in the lower panel (Bonferroni test). A P<0.05 was considered statistically significant.

| Group | Birth | Weaning | 70 days | Slaughter |
| --- | --- | --- | --- | --- |
| 1 | Reference group | 2.3 (1.4) | 27.3 (1.1) | 97.1 (1.1) |
| 2 | 0.5 (1.4) | 5 (1.6) | 27.3 (1.4) | 97.1 (1.4) |
| 3 | 1.1 (1.6) | 4.4 (1.7) | 28.4 (1.6) | 100.5 (1.6) |
| 4 | - 1. (1.6) | 6.4 (1.7) | 27.6 (2) | 103.4 (1.6) |
|  |  |  |  |  |
| Groups compared | P value | P value | P value | P value |
| 1-2 | P=0.700 | P=0.150 | P=0.999 | P=0.998 |
| 1-3 | P=0.470 | P=0.305 | P=0.517 | P=0.035 |
| 1-4 | P=0.467 | P=0.040 | P=0.871 | p<0.001 |
| 2-3 | P=0.681 | P=0.745 | P=0.483 | P=0.021 |
| 2-4 | P=0.664 | P=0.427 | P=0.855 | p<0.001 |
| 3-4 | P=0.983 | P=0.257 | P=0.575 | P=0.045 |

**Table S3:** **number of dead, weak and tail bitten piglets/pigs**

Descriptive statistics for piglets'/pigs' condition, from age of three days to slaughter. Data is presented as the number of animals and the corresponding percentages (in brackets). Chi-square goodness of fit of “weak” among all four Groups: X^2^_(3)_=18.059, P<0.001. Mixed effects binomial test among the dead piglets/pigs: X^2^_(1)_=4, P<0.031. ^a,b,c^Values within a columns with different letters differ significantly (P <0.05).

| Tail  bitten | Weak or dead | Total dead | Dead before slaughter | Dead before weaning | Weak | Group size (n) | Group |
| --- | --- | --- | --- | --- | --- | --- | --- |
| 4  (4.5%) | 20  (22.7%)^a^ | 13 (14.7%)^a^ | 4  (4.5%) | 9  (10.2%) | 10  (11.3%)^a^ | 88 | **1** |
| 2  (2.4%) | 15  (18.2%)^ab^ | 4  (4.9%)^b^ | 1  (1.2%) | 3  (3.7%) | 7  (8.5%)^a^ | 82 | **2** |
| 0  (0%) | 7  (8.9%)^ab^ | 7  (8.9%)^b^ | 2  (2.6%) | 5  (6.4%) | 0  (0%)^b^ | 78 | **3** |
| 0  (0%) | 4  (4.9%)^b^ | 4  (4.9%)^b^ | 1  (1.2%) | 3  (3.7%) | 0  (0%)^b^ | 81 | **4** |

**Table S4: Odds ratios of piglets to be weak or die in Groups 2-4, as compared to Group 1**

Data was analyzed by mixed effects logistic regression; Random effect: mother. Predictor: treatment. Analysis was adjusted for: Piglet Sex, Sows’ cycle number, number of raised piglets (alive piglets on the day performing invasive procedures) and suckling period.

| Group | Odds Ratio | Confidence Interval | P value |
| --- | --- | --- | --- |
| 1 | Reference group |  |  |
| 2 | 0.47 | 0.11-1.86 | P=0.280 |
| 3 | 0.34 | 0.08-1.4 | P=0.137 |
| 4 | 0.11 | 0.02-0.68 | P=0.015 |

**Table S5:** **Injuries of pigs** **in Groups 2-4, as compared to Group 1.**

Incident data of injuries was analyzed by a Mixed effects poisson regression coefficient, for groups 2-4 as compared to the reference group, Group 1.

| Group | B | Confidence Interval | P value |
| --- | --- | --- | --- |
| 1 | Reference group |  |  |
| 2 | -0.405 | -0.78,-0.031 | P=0.034 |
| 3 | -0.824 | -1.234,-0.414 | P=0.000 |
| 4 | -0.693 | -1.068,-0.319 | P=0.000 |

**Table S6: Saliva Cortisol in Groups 2-4, as compared to Group 1.**

Saliva cortisol was evaluated every two weeks from weaning to slaughter at the pen level. Data was analyzed by linear regression model for Groups 2, 3, and 4 as compared to Group 1.

| Model | β | SEM | P Value |
| --- | --- | --- | --- |
| Group 1 | Reference group |  |  |
| Group 2  Group 3  Group 4 | -0.86  -1.3  -0.71 | 0.28  0.45  0.15 | 0.008  0.011  0.000 |

B- (ln) saliva cortisol coefficients

**Table S7: Cost items by rearing method**

|  | **Conventional rearing** | | | | **Welfare-friendly rearing** | | | | **Source** |
| --- | --- | --- | --- | --- | --- | --- | --- | --- | --- |
| **Items** | **Cost** | **Unit** | **Per** | **Country** | **Cost** | **Unit** | **Per** | **Country** |  |
| **Labor** | 0.0625 | hours | Head | Israel | 0.005556 | Hour | Head | Israel | Morgan et al.* |
| **Salary** | 1.848125 | $ | Head | USA | 0.164278 | $ | Head | USA | Morales J, et al.[^1^](#_ENREF_1) |
| **Vaccine** | - | - | - | - | 1.6648 | $ | Male | Israel | Morgan et al. |
| **Lidocain** | 0.005 | Euro | Male | UK | - | - | - | - | de Roest et al. [^2^](#_ENREF_2) |
| **Meloxican** | 0.14 | Euro | Head | UK | - | - | - | - | de Roest et al. [^2^](#_ENREF_2) |
| **Equipment** | 1,788 | $ | Year | Israel | - | - | - | - | Morgan et al. |
| **Weight** | 97.1 | kg | Head |  | 103.4 | kg | Head |  | Morgan et al. |
| **Mortality** | 0.147727 | Rate |  |  | 0.049383 | Rate |  |  | Morgan et al. |
| **Extra Raising** | 17.72727 | $ | Head |  | 5.925926 | $ | Head |  | Morgan et al. & O'Neel [^3^](#_ENREF_3) |

*Morgan et al. means the current study

**Table S8**: Demand and supply elasticities based on the literature

| **Study** | **Years** | **Supply elasticity** | **Demand elasticity** |
| --- | --- | --- | --- |
| Dean and Heady 1958 [^4^](#_ENREF_4) | 1924-1956 | 0.28 to 0.6 | between -0.62 and -2.75 |
| Tweeten 1970 [^5^](#_ENREF_5) | - | 0.6 | - |
| Lemieux and Wohlgenant 1989 [^6^](#_ENREF_6) | 1955-1983 | (1-year) 0.4; (5-year) 0.6 | (used) -0.7 -0.9 |
| Wade and Barkley 1992 [^7^](#_ENREF_7) | 1959-1989 | (short) 0.23; (long) 0.391 | -2.545 |
| Dahlgran 1987 [^8^](#_ENREF_8) | 1960's  1985 | -  - | -0.914  -0.584 |

**Figure S1:** **environmental enrichment during nursing and fattening periods.**

Both cotton ropes and jute strips were hung on crates/pen bar, starting a few days before anticipated farrowing, and were refilled during the whole nursing period. From weaning until the age of 70 days, cotton ropes and Bite-Rite® chewable silicon devices (Ikadan System, Denmark) were provided; cotton ropes and chewable silicon sticks of the Bite-Rite® devices were maintained constantly. From the age of 70 days to slaughter, environmental enrichment included straw, which was provided in commercial grubbing straw racks (Domino, Denmark) designed for pigs, as well as Bite-Rite® devices.


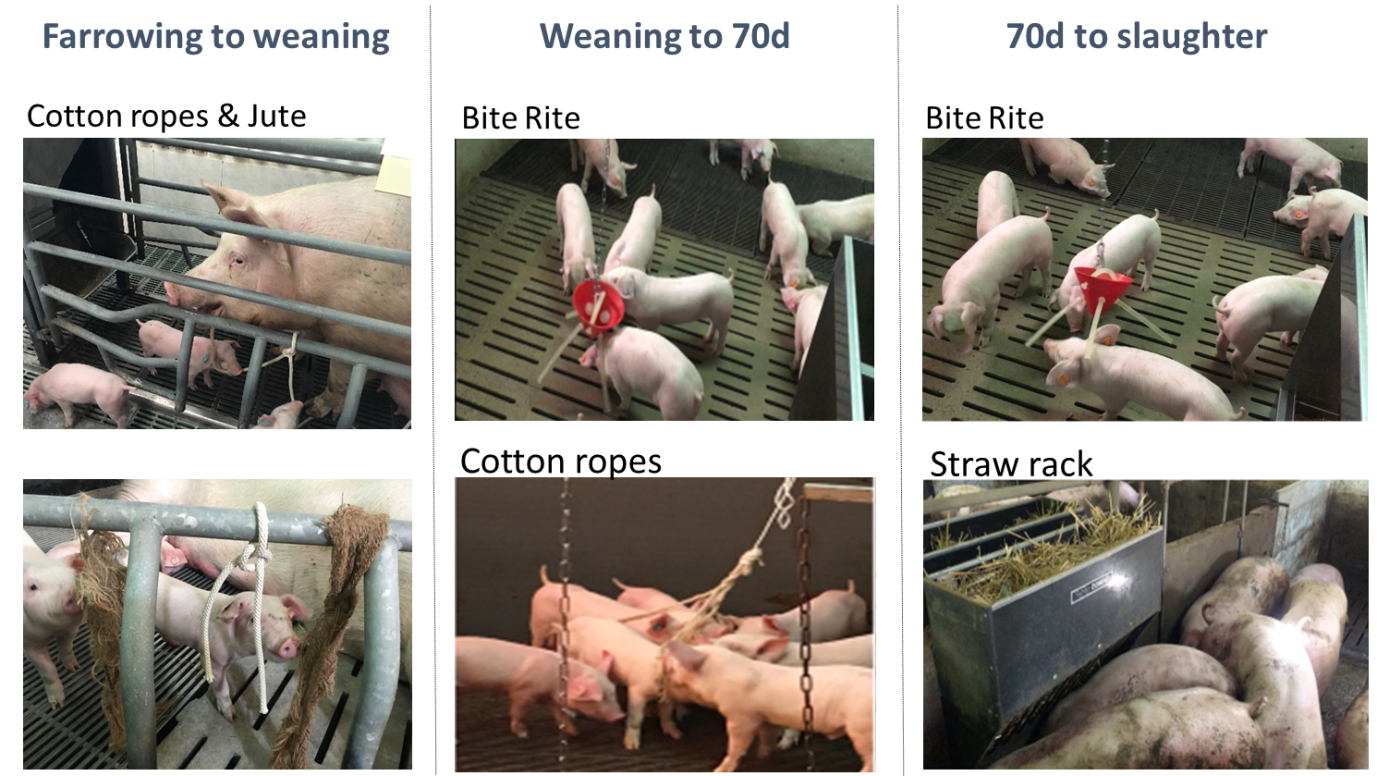


# **References**

1 Morales, J. *et al.* Surgical castration with pain relief affects the health and productive performance of pigs in the suckling period. *Porcine Health Management* **3**, doi:10.1186/s40813-017-0066-1 (2017).

2 de Roest, K., Montanari, C., Fowler, T. & Baltussen, W. Resource efficiency and economic implications of alternatives to surgical castration without anaesthesia. *Animal : an international journal of animal bioscience* **3**, 1522-1531, doi:10.1017/S1751731109990516 (2009).

3 O'Neel, T. Pork Checkoff Annual Report. Available at: https://[www.pork.org/wp-content/uploads/2018/2003/Checkoff-Annual-Report.pdf](http://www.pork.org/wp-content/uploads/2018/2003/Checkoff-Annual-Report.pdf) [accessed November 2018] (2017).

4 Dean, G. W. & Heady, E. O. Changes in Supply Response and Elasticity for Hogs. *American Journal of Agricultural Economics* **40**, 845-860, doi:10.2307/1234771 (1958).

5 TWEETEN, L. (Lincoln, Nebr., USA: Univ. Nebr. Press., 1970).

6 Lemieux, C. M. & Wohlgenant, M. K. "Ex Ante" Evaluation of the Economic Impact of Agricultural Biotechnology: The Case of Porcine Somatotropin. *American Journal of Agricultural Economics* **71**, 903-914, doi:10.2307/1242668 (1989).

7 Wade, M. A. & Barkley, A. P. The economic impacts of a ban on subtherapeutic antibiotics in swine production. *Agribusiness* **8**, 93-107, doi:doi:10.1002/1520-6297(199203)8:2<93::AID-AGR2720080202>3.0.CO;2-9 (1992).

8 Dahlgran, R. A. Complete Flexibility Systems and the Stationarity of U.S. Meat Demands. *Western Journal of Agricultural Economics* **12**, 152-163 (1987).
